# STARS: Creating robust infrastructure to scale up structural biology programs for students

**DOI:** 10.1063/4.0000843

**Published:** 2025-10-27

**Authors:** Susanna Huang

**Affiliations:** 1 Georgia Institute of Technology, Atlanta, GA, USA; 2 Structural Nucleic Acid Anticancer Research Society (STARS), Atlanta, Georgia, USA

## Abstract

Structural biology plays a key role in therapeutic drug discovery by revealing mechanisms of drug leads against their macromolecular targets and allowing for the optimization of their structures to enhance their therapeutic properties. One way that structural biology has been widely performed is by crystallography, which encompasses protein expression, crystallization, diffraction data collection, and model building and refinement. While experiments employing these techniques often are hard for students to perform on their own, since they require steep expertise, equipment, and funding barriers, the crystallographic and adjacent experiences can offer students unique opportunities to learn valuable research skills that they can use for projects they may want to pursue in the future in an engaging, captivating manner.

The Structural Nucleic Acid Anticancer Research Society (STARS) is a student-led nonprofit organization that aims to provide these educational opportunities in relatable and engaging ways to students by creating robust infrastructure needed to support K-8th educators, 9th-12th graders, and undergraduate students to implement inorganic crystal-growing and macromolecular crystallography club projects and outreach programs to provide valuable experiences and skills, such as micro pipetting solutions for experimental setups, analyzing molecular interactions in protein complexes, or practice diffracting crystals, to elevate students’ understanding and learning of crystal-growing and crystallography along with key scientific skills, such as observation, notetaking, and analysis skills, so that students may use these experiences for their own projects in the future.

Our strategy is to provide club branch projects, which can have therapeutic significance, that (1) provide valuable scientific experiences to students; (2) are applicable to students’ research interests; (3) are thematically accessible and easily understandable in nature; and (4) are rendered much more feasible to perform through clear and concise handouts provided by STARS and through branch fundraising with clear budgets. In starting new STARS branches in K-12th and in undergraduate student populations, we aim to not only equip students with important scientific skills and experiences through club branch activities, such as crystal-growing or crystallography projects, and outreach programs, such as crystallography workshops and crystal-growing competitions, but also aim to build up student leaders in science by providing students the opportunity to advocate for their STARS branch mission and purpose, share their outreach and research message through presentations, and network with industry and academia leaders at, for example, the American Crystallographic Association conference.

So far, STARS already has two STARS branches, where student leaders bring STARS programming and outreach to life. These two branches have together organized over 12 events and programs, such as crystal-growing competitions, crystallography workshops, and lecture series sessions over the past four years with 380+ participants cumulatively. However, to truly provide all students in the United States the crystal- growing and crystallography opportunities to learn valuable scientific skills and be inspired in research for the treatment of diseases, STARS aims to (1) fully streamline its club programming and outreach activities with clear guidance and handouts, which can enable our STARS programs to scale up; (2) foster inter-STARS branch communications and collaborations to form a network of research-focused students through our annual STARS meetings; (3) enable students more accessible opportunities to give presentations at the American Crystallographic Association (ACA) conference through the STARS Travel Grant; and (4) engage more K-12th and undergraduate students in not only inorganic crystal growing, but also protein crystallography through national crystallography competitions of proteins (such as with lysozyme, the chicken egg white protein).

Crystallography is a unique research field that not only is very important for structural biology and therapeutic drug discovery research but also can easily captivate students with its beautiful inorganic and macromolecular crystals and structures. The Structural Nucleic Acid Anticancer Research Society (STARS) aims to capitalize on these inherent qualities to engage and empower students in crystal-growing, crystallography, and therapeutic research through our streamlined programs and club activities.

